# Provenance and risk in transfer of biological materials

**DOI:** 10.1371/journal.pbio.2006031

**Published:** 2018-08-13

**Authors:** Jane Nielsen, Tania Bubela, Don R. C. Chalmers, Amber Johns, Linda Kahl, Joanne Kamens, Charles Lawson, John Liddicoat, Rebekah McWhirter, Ann Monotti, James Scheibner, Tess Whitton, Dianne Nicol

**Affiliations:** 1 Centre for Law and Genetics, Faculty of Law, University of Tasmania, Hobart, Tasmania, Australia; 2 Faculty of Health Sciences, Simon Fraser University, Burnaby, Canada; 3 Cancer Division, Garvan Institute of Medical Research, Darlinghurst, New South Wales, Australia; 4 BioBricks Foundation, San Francisco, California, United States of America; 5 Addgene, Cambridge, Massachusetts, United States of America; 6 Griffith Law School and Australian Centre for Intellectual Property in Agriculture, Griffith University, Gold Coast, Queensland, Australia; 7 Faculty of Law, University of Cambridge, Cambridge, United Kingdom; 8 Faculty of Law, Monash University, Clayton, Victoria, Australia

## Abstract

Whereas biological materials were once transferred freely, there has been a marked shift in the formalisation of exchanges involving these materials, primarily through the use of Material Transfer Agreements (MTAs). This paper considers how risk aversion dominates MTA negotiations and the impact it may have on scientific progress. Risk aversion is often based on unwarranted fears of incurring liability through the use of a material or loss of control or missing out on commercialisation opportunities. Evidence to date has suggested that complexity tends to permeate even straightforward transactions despite extensive efforts to implement simple, standard MTAs. We argue that in most cases, MTAs need do little more than establish provenance, and any attempt to extend MTAs beyond this simple function constitutes stifling behaviour. Drawing on available examples of favourable practice, we point to a number of strategies that may usefully be employed to reduce risk-averse tendencies, including the promotion of simplicity, education of those engaged in the MTA process, and achieving a cultural shift in the way in which technology transfer office (TTO) success is measured in institutions employing MTAs.

## Introduction

A broad range of biological materials are transferred between laboratories to keep these engine rooms of innovation ticking. Material Transfer Agreements (MTAs) are common vehicles for exchanging these materials, whether between laboratories in universities and research institutes or between these organisations and industry [1]. Convoluted negotiations between institutional technology transfer offices (TTOs) tend to dominate even straightforward MTA transactions [2,3,4]. Indiscriminate use of complicated MTAs could unnecessarily slow progress in the biological sciences, given that very few are ever likely to be enforced [2,3]. Our collective experience leads us to conclude that a) the main purpose of written MTAs should not be to ascribe ownership but simply to establish provenance, the pathway the material takes from its point of origin; and b) the core problem that interferes with achievement of a) is unreasonable risk aversion.

MTAs provide clear ‘chains of custody’ by documenting the paths travelled by materials [5], recording their origins and encumbrances imposed by previous agreements (including funding agreements), and listing other rights and obligations attaching to them. While MTAs serve a useful purpose, negotiations due to perceived risks can delay transfers and impose research impediments [4]. Extended negotiations are often based on overestimated fears of incurring liability through use, loss of control, or missing out on speculative commercialisation opportunities [3].

One step that has been taken to streamline MTA processes is the implementation of standard form agreements [6]. The Simple Letter Agreement (SLA) and Uniform Biological Material Transfer Agreement (UBMTA), developed by the National Institutes of Health (NIH) and the Association of University Technology Managers (AUTM), are a well-documented attempt to achieve the goal of simplifying exchanges between universities and research institutes. The number of signatories to the UBMTA suggests that there is widespread support for the implementation of this initiative. Despite this, many parties are unable to resist the temptation to tweak agreements, which compels organisations receiving them to carefully examine them for discrepancies, variations, or anything perceived to disadvantage the interests of their own institution [1,4,7].

Once we accept that MTAs are a necessary part of the exchange process and that simplifying transfers would increase research efficiency and be a more efficient use of administrative resources, the challenge is to determine how to improve MTA practices.

## Examples of good practice

Structural genomics and computational biology represent areas of research in which there has been a longstanding commitment to principles of open access. Openness is regarded as technologically important in these fields because of a need to ensure interoperability of bioinformatics software and promote the development of common ontologies for the virtual expression of biological concepts. This is evidenced by the large number of bioinformatics software packages that are available under open source licences and on online open source repositories.

The adoption of open access principles has been less straightforward in other fields. Even within initiatives specifically designed to facilitate exchanges of materials for research purposes, there is evidence that parties can still unduly complicate the transfer process. An example is the Knockout Mouse Project, in which European repositories utilised restrictive terms for deposit and distribution, flowing, in part, from concerns about use, appropriate attribution of the source of material, and distribution of profits from commercial exploitation [8]. This led to frustration and delays rather than systemic failure of the initiative.

Other models have achieved greater success, as illustrated by the examples provided in Boxes 1 and 2.

**Box 1. Addgene**

Research utilising Clustered Regularly Interspersed Short Palindromic Repeats (CRISPR) is providing a significant advance in the development of accurate and safe editing of the genome of all living organisms. The development of CRISPR technology is being facilitated by open distribution of CRISPR reagents, RNA, and plasmids for academic research use through Addgene, a nonprofit intermediary established specifically for the purpose of accelerating science by providing access to materials and information [12].

**Fig 1:**
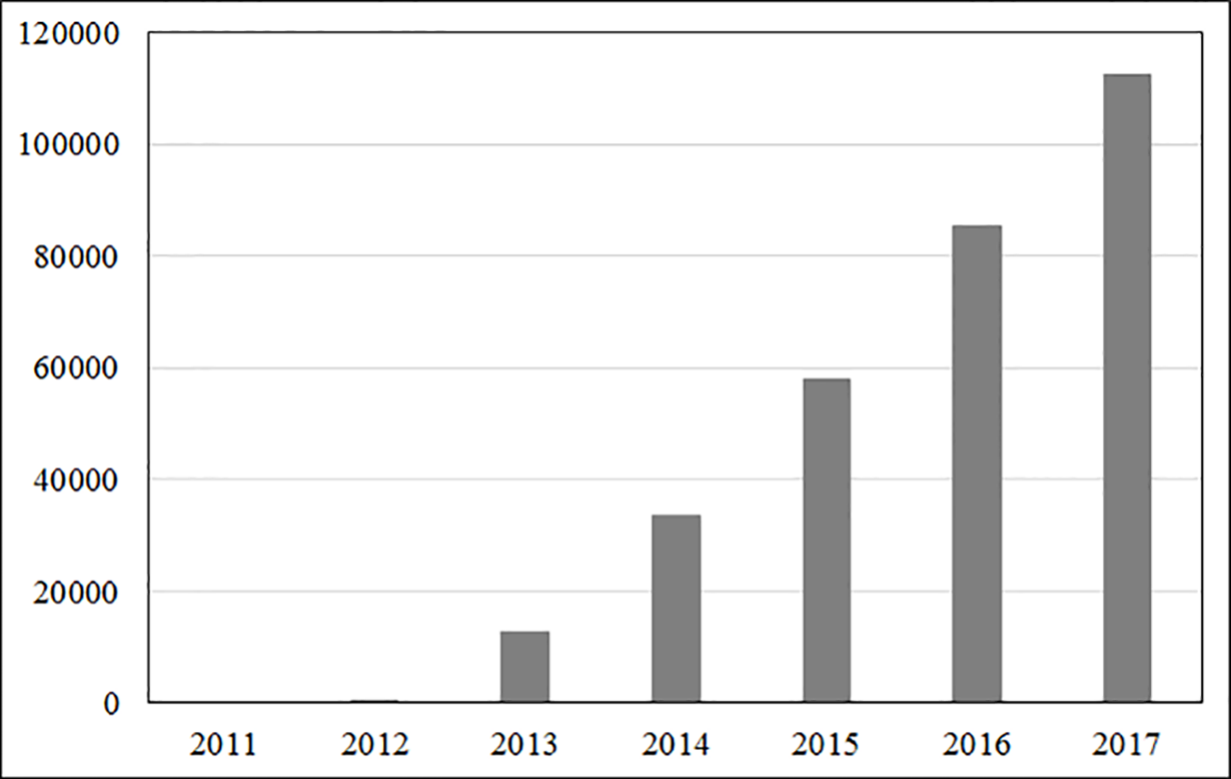
Distribution of CRISPR-Related Plasmids (number) by Addgene (cumulative).

The process for distribution of CRISPR constructs via Addgene starts with the depositing scientist’s TTO signing a deposit agreement, which authorises Addgene to distribute the materials under a standard UBMTA. The requesting scientist’s TTO is required to complete the MTA and most often does so via Addgene’s custom electronic MTA (eMTA) signature system. The average turnaround time for Addgene’s eMTA is 2 days. In this way, one signature can translate into thousands of rapidly executed MTAs. One of the most important features of the system is the fact that Addgene keeps detailed and accurate records and makes these transaction records available via online accounts to both materials depositors and their TTOs. The capability of knowing where all one’s materials have been distributed is a primary benefit to such a system and one of the most important aspects of MTA completion. Addgene has facilitated over 200,000 MTAs. Addgene is currently distributing materials under the UBMTA terms at a rate of over 600 items daily to scientists in over 95 countries. Fig 1 illustrates how demand for CRISPR constructs through Addgene has grown exponentially.

Addgene has successfully provided a process between researchers to facilitate simple, efficient, and accurately recorded exchanges of CRISPR materials [13]. However, there is one note of caution in that not all CRISPR constructs are available through Addgene. This illustrates a potential difficulty of encouraging basic research for fields with complicated commercial interests [14], particularly for transformative technologies in which academic and commercial interests collide. It does not necessarily have to be the case that concurrent transfer of relevant materials for academic research purposes must cease once there is a commercially promising lead.

**Box 2. BioBricks, OpenMTA, and iGEM**

For more than a decade, the BioBricks Foundation (BBF) has developed legal tools and frameworks to provide options for synthetic biologists and life science researchers generally to work in a more open, collaborative, and distributive manner. The BBF’s flagship legal tool—the BioBrick Public Agreement, version 1.0 (BPAv1.0)—provides a framework for making engineered biological sequences available for others to use. Sequences contributed under BPAv1.0 may be accessed as information through the BBF’s website, thereby enabling those with access to DNA synthesis capabilities to create physical biomaterials based on these sequences. In addition, some of the sequences contributed under BPAv1.0 have been incorporated into plasmids that may be accessed through Addgene (https://www.addgene.org/biobrick-public-agreement-collection/).

More recently, the Open Material Transfer Agreement (OpenMTA) was developed to specifically address the tangible rights inherent in sequences shared as physical biomaterials. The goal was to develop a simple, standardised MTA for sharing biological materials as broadly as possible and without restriction while honouring the rights of others and promoting safe laboratory practice and responsible research. Development of the OpenMTA was conducted as a collaborative effort led by the BBF and the OpenPlant Synthetic Biology Research Centre with input from researchers, social scientists, technology transfer professionals, legal experts, business leaders, and government and funding agencies.

Because the OpenMTA is constructed as a standard template, it can easily be incorporated into semiautomated MTA administration systems, such as Addgene’s eMTA signature system. This will help speed and simplify MTA processing while providing a less restrictive option for material transfer when appropriate. As of this writing, the formal review period for the OpenMTA Master Agreement is still open. Information about the OpenMTA—including design goals, FAQs, and videos—can be accessed at OpenMTA.org.

The iGEM Foundation, or the International Genetically Engineered Machine (iGEM), houses a Registry of Standardised Biological Parts, which serves as the primary resource of biomaterials. The Registry now has over 20,000 biological parts and is growing each year. DNA sequence and other information about the biological parts in the Registry is available online (http://parts.igem.org/Main_Page). In addition, the iGEM Foundation shares biological parts from the Registry with academic laboratories upon request. As is the case for the student competition, the iGEM Foundation shares biological parts with academic labs without an MTA in place.

A requirement for biological parts to be submitted to the Registry is that the parts must be compatible with the BioBrick RFC10 assembly standard, which is a technical standard that enables the parts to be easily combined with one another. Today, the two organisations continue to work in partnership to support open, collaborative, and responsible innovation in the field of synthetic biology.

What lessons can we take from these examples in shaping strategies to facilitate cultural change and reduce perceptions of risk that may ultimately hinder the process of transfer? First, the use of distribution networks and intermediaries creates efficiencies by removing choice from the negotiating process, as illustrated by the Addgene example. Access to material via Addgene is contingent on execution of the simple online agreement by the requesting institution, leaving no room for negotiation of individual terms. Contrast this with the Knockout Mouse Consortium, in which room for individualisation remained. Another feature of such arrangements, as illustrated by BioBricks, is reduction in the number of MTAs required for a particular research project.

Second, the emphasis on ‘openness’ that is a hallmark of the open software movement, from which open bioinformatics initiatives have emerged, can be translated into other areas of biotechnology [9]. The OpenMTA is a simple, standardised agreement that enables researchers to exchange biomaterials more freely, with features such as attribution, provenance tracking, and ease of use internationally to work within the practical realities of technology transfer [10].

Third, involvement in large-scale research collaborations like BioBricks builds good relationships and trust. It also aligns motivations of the parties involved. While not every TTO will have experience in MTAs associated with large-scale collaborations, lessons can be learned from examining the fundamental bases for the success of these larger networks in facilitating free-flowing exchanges. Parties undertaking research with mutually beneficial outcomes are less likely to strategize at the expense of reaching rapid agreement. Again, lack of choice is a feature; the terms on which materials are exchanged are fixed, removing the opportunity for TTOs to modify.

How might we translate these lessons into the world of bilateral, institutional material transfers? Promoting exchange of materials on standardised, simple terms is an important first step. The real challenge lies in finding a way to implement these lessons to incorporate simplicity more broadly in entity-to-entity MTAs.

## When is complexity needed?

There are undoubtedly circumstances where an escalation to complexity is necessary; for instance, when commercial parties are involved and the purpose of the exchange goes beyond research use only [3,4]. This distinction between research transfers and transfers of a commercial nature is reflected in the Addgene arrangements, which include separate considerations for industry recipients and, in accordance with the UBMTA, require written consent of the provider for commercial use by the recipient. Many commercial parties, particularly in the drug-development space, are likely to make significant investment into research activities involving licensed materials and will expect some ongoing control over research outcomes and profits. Institutional MTAs with commercial endpoints also warrant specificity with regard to authorship and acknowledgment, reservations of rights to use materials on a continuing basis, and allocation of profits.

There are other situations in which the nature of the material renders simplified provenance documentation unsuitable, even when the material is exchanged for research purposes. This is not to say that standardised agreements cannot be used for similar types of materials, but their unique qualities might require the application of individual standards. Box 3 illustrates applicable cases. An example is research involving materials that will lead to a clinical outcome: clear parameters will be required in MTAs of this nature because of ethical and benefit-sharing issues. These issues underscore exchanges of materials provided by indigenous donors.

**Box 3. Tissue sourced from indigenous peoples**

Sharing of tissue samples collected from indigenous participants with other researchers may be complicated by the inclusion of culturally specific conditions within consent agreements. Appropriately designed MTAs may facilitate culturally sensitive research practices by enforcing consent provisions on downstream research use of the samples. Conversely, standardised MTAs that preclude culturally specific provisions may lead to the exclusion of indigenous groups from genetic research, either by making it too difficult to share tissue samples or by compromising trust in the research endeavour, thereby adversely affecting indigenous participation rates. Exclusion of indigenous populations from genetic research reduces the generalisability of resultant genomic medicine and may exacerbate persistent health inequalities [15].

**United Kingdoms Biobank**

United Kingdoms Biobank was established in 2007 by a range of organisations, including the Wellcome Trust and the UK Department of Health. It was established as a nonprofit charity and had initial funding of approximately UK£62 million. It is hosted by the University of Manchester and supported by the UK National Health Service. UK Biobank has not been designed as a ‘research project’ but rather as a key piece of scientific infrastructure for life sciences research. UK Biobank collected biological samples from 500,000 people aged between 40 and 69 years of age. Between 2012 and 2016, 267 projects were approved for access to UK Biobank. Research groups can gain access to UK Biobank via an authorisation process and payment of a charge. The charge is based on a cost-recovery basis. Authorisation does not distinguish between commercial and noncommercial research but does review the scientific validity of a project and whether it is in the public interest. Authorisation, amongst other things, also reviews an applicant’s ability to store and ensure the security of materials (including data). Authorised researchers are granted limited licences to use the UK Biobank resource, and these rights are not assignable. When researchers create additional datasets as a result of using the UK Biobank resource, then intellectual property (IP) in these datasets is owned by the researchers (or their institutions) [16]. Researchers are required to submit these datasets to UK Biobank within six months following their publication, or within 12 months following completion of the research project. Other IP developed using the UK Biobank resource is free from any other obligation to UK Biobank, except in one circumstance: if use of the IP rights is deemed ‘unreasonably restrictive’ by UK Biobank, then UK Biobank reserves the right to require that the IP is licensed to UK Biobank on a royalty-free, sublicensable, and nonexclusive basis.

**Transfers of sovereign genetic resources**

The United Nations Convention on Biological Diversity (CBD) effectively established a global framework for the exchange of nonhuman biological materials requiring prior informed consent, mutually agreed terms, and equitable benefit sharing negotiated as part of the mutually agreed terms. This is satisfied by a traditional contractual arrangement between the resource provider and the party access in the form of a materials transfer agreement. Some of the nations complying with these obligations have implemented their domestic laws providing for these contracting requirements. A more comprehensive Nagoya Protocol on Access to Genetic Resources and the Fair and Equitable Sharing of Benefits Arising from their Utilisation to the Convention on Biological Diversity (Nagoya Protocol) has also been agreed setting out the mechanisms for these contractual and benefit sharing arrangements. As might be expected, however, only a few nations have implemented the required CBD and Nagoya Protocol laws, and the expected benefits flowing from the benefit sharing agreements have not eventuated.

More recently, agreements directed to some agriculturally important crop plants under the Food and Agriculture Organisation International Treaty on Plant Genetic Resources for Food and Agriculture (Plant Treaty) and human pandemic influenza viruses under the World Health Organisation Pandemic Influenza Preparedness Framework (PIP Framework) have been implemented and provide for Standard Material Transfer Agreements (SMTAs) with set terms and conditions of exchanges.

Exchanges mediated by biobank intermediaries are also complicated by the fact that participants may be asked to consent not just to individual research projects that use their material (as is normally the case) but more generally to uses that fall into the overarching purpose for which the biobank was established. Access to biobank resources (such as the UK BioBank and similar arrangements) for research purposes thus needs careful management, going beyond the terms of generic MTAs. Another area of particular concern is the exchange of nonhuman materials sourced from natural resources, for which benefit sharing is paramount. The CBD and Nagoya Protocol attempt to provide an international framework to facilitate such exchanges.

These examples are not exclusive but illustrate the point that there are justifiable reasons for increasing the complexity of MTAs in some circumstances. In purely bilateral transactions, there is considerably more scope for institutional idiosyncrasies to overtake simplicity if risk aversion is allowed to dominate the transfer process. Many continue to adhere to a culture protecting against ‘missing out’ rather than facilitating open exchanges.

## Understanding risk to overcome unreasonable aversion

Realistically, very few MTAs are likely to be monitored, and the likelihood that a risk-averse institution would embark on the highly risky course of litigation to enforce an MTA is remote [3]. In light of this, how might we encourage TTOs to acknowledge that simple transfer procedures will be appropriate in the majority of material transfers? In order to implement real change, the expectations of TTO personnel and the format of MTAs must change concurrently. This ‘combination’ of approaches has driven the success of initiatives such as Addgene, the BioBricks Foundation, and the iGEM Foundation. Under these arrangements, simplicity is essential. MTAs are standardised and simplified, and institutional adherence to these standards is mandated.

The success of Addgene, BioBricks and iGEM can in part be attributed to the common interests and goals of participants—to minimise delay in research projects that require access to research materials on reasonable terms. These common interests, originating in the open source movement, have also driven some of the more complex arrangements for transfer of materials for research purposes. The UK Biobank, for example, has developed strategies to deal with the complexities associated with ethics, consent, and benefit sharing and overcome risk aversion.

One important aspect of each of these initiatives is that they have a single gatekeeper who plays a vital role in preventing deviations. The difficulty that universities and research institutes face is that, unlike these initiatives, there is no single body that can dictate the form of MTA that is to have universal application for simple transfers and to manage the transfer process. Instead, multiple institutions must be persuaded to adopt a common approach to practices and procedures in the execution of MTAs. This requires them to recognise deficiencies in the status quo. The move to standardise MTAs in the context of transfers of sovereign genetic resources led by the United Nations, the Food and Agriculture Organisation, and the World Health Organisation provides an example of how institutions can be drivers of change. On a different scale, it is open to national public research funding organisations to condition research grants on dissemination of materials using simplified processes. The NIH and AUTM have been particularly proactive in this regard [11].

There are a number of strategies that may reduce friction in MTA negotiations and expedite the MTA process. Applying simplicity during the process of negotiating for the transfer of materials is crucial to eradicating risk-averse behaviour. This includes, where possible, the removal of choice in the application of MTAs. Educating those involved in negotiating and drafting MTAs is fundamental, particularly through utilisation of the expertise of representative bodies.

Promoting a shift in the way that ‘success’ in material transfers is perceived within institutions is also essential. TTO personnel possess a surprisingly social view of their roles in promoting the research agendas of their institutions [6]. This supports the proposition that change in culture is possible, and one option for encouraging this is to invert current notions of risk aversion for publicly funded institutions. Rather than focusing on the perceived risks that TTOs identify in association with exchange of materials, the ‘risk’ against which TTO performance should be measured is that of nonconformity with conceptions of open sharing.

## Supporting information

S1 Data S1 Data. (XLSX)

## Abbreviations

AUTM: Association of University Technology Managers
BBF: BioBricks Foundation
BPAv1.0: BioBricks Public Agreement, version 1.0
CBD: Convention on Biological Diversity
CRISPR: Clustered Regularly Interspersed Short Palindromic Repeats
eMTA: electronic Material Transfer Agreement
iGEM: International Genetically Engineered Machine
IP: intellectual property
MTA: Material Transfer Agreement
NIH: National Institutes of Health
OpenMTA: Open Material Transfer Agreement
PIP: Pandemic Influenza Preparedness
SLA: Simple Letter Agreement
SMTA: Standard Material Transfer Agreement
TTO: technology transfer office
UBMTA: Uniform Biological Material Transfer Agreement
